# Airway hygiene in the ICU: can ipratropium plus salbutamol help?

**DOI:** 10.1186/cc14289

**Published:** 2015-03-16

**Authors:** N Magalhaes

**Affiliations:** 1 Centro Hospitalar Tâmega e Sousa, Penafiel, Portugal

## Introduction

β-Adrenergic agonists increase the ciliary beat frequency in experimental models, raising the possibility that they may be useful for airway hygiene [1]. Salbutamol increases large airway mucociliary clearance [2], although this may not be true for smaller airways [3]. There are no data from ICU patients, so we decided to test the effectiveness of transtracheal instillation of a mixture of ipratropium and salbutamol, assessing the reduction of the number of aspirations and hours of ventilation.

## Methods

An open randomized prospective study was held during 2014. All admitted patients were alternately selected as the study or control group and included if submitted to invasive ventilation for at least for 24 hours. Four patients who had secondary cardiac rhythm effects attributable to β-adrenergic agonists were excluded. In the study group, 3 ml of a dilution of 2.5 ml ipratropium (0.52 mg) plus salbutamol (2.5 mg) with 2.5 ml distilled water was instilled after the aspiration of secretions. During the ventilation period we noted for both groups, in addition to the demographic data, the number of tracheal aspirations by day and hours of ventilation. The statistical analysis was done with XLSTAT 2014 and we used the Kolmogorov- Smirnov test to compare the normal distribution of the groups.

## Results

These preliminary results included 107 patients. The only normal distribution, according to the Kolmogorov-Smirnov test, was for the number of hours of ventilation: 76.3 ± 100 (24; 478) in the study group versus 111 ± 167 (24; 780) in the control group.

## Conclusion

The preliminary analysis, referring to the first 6 months of study, showed a tendency in the reduction of both ventilation hours and length of stay in the study group. No significant difference was found in the number of aspirations, which may be explained by ICU nursing routines. Further studies will try to find whether significant differences in the incidence of VAP exist, allowing this procedure to be implemented in ICU routines.
